# Multirobot Heterogeneous Control Considering Secondary Objectives

**DOI:** 10.3390/s19204367

**Published:** 2019-10-09

**Authors:** Julio F. Acosta, Guillermo González de Rivera, Víctor Hugo Andaluz, Javier Garrido

**Affiliations:** 1 Universidad de las Fuerzas Armadas–ESPE, Sangolquí 171103, Ecuador; vhandaluz1@espe.edu.ec; 2 Escuela Politécnica Superior, Universidad Autónoma de Madrid, 28049 Madrid, Spainjavier.garrido@uam.es (J.G.)

**Keywords:** heterogeneous robots, control based on linear algebra, cooperative control, control considering secondary objectives

## Abstract

Cooperative robotics has considered tasks that are executed frequently, maintaining the shape and orientation of robotic systems when they fulfill a common objective, without taking advantage of the redundancy that the robotic group could present. This paper presents a proposal for controlling a group of terrestrial robots with heterogeneous characteristics, considering primary and secondary tasks thus that the group complies with the following of a path while modifying its shape and orientation at any time. The development of the proposal is achieved through the use of controllers based on linear algebra, propounding a low computational cost and high scalability algorithm. Likewise, the stability of the controller is analyzed to know the required features that have to be met by the control constants, that is, the correct values. Finally, experimental results are shown with different configurations and heterogeneous robots, where the graphics corroborate the expected operation of the proposal.

## 1. Introduction

Recent advances in robotics have allowed the migration from the operation of a single large vehicle to the integration of many ones in search of a common goal. This migration is the result of optimizing various processes, which require a robot with great capabilities to a set of robots with distinctive features. Applications such as the search and rescue of objectives in natural disasters [1], displacement of extremely heavy or very long objects, mapping of land [2], assembly of pieces, and so on, are an example of the need to bring together small-scale robots and lead them on a common task [3]. In this type of terrestrial applications, the mobile robots based on wheels predominate over robots composed of legs or caterpillars, subdividing them in the way in how the wheels are distributed in the mechanism [4,5].

The usual configuration of mobile robots consists of two wheels joined by a common axis, where the stability of the mechanism is achieved by placing a castor-type wheel. This type of vehicle has had a considerable amount of analysis given the ease of its construction, despite its limitations, such as the non-holonomicity (a common feature of wheeled robots in general) or the loss of traction in sharp turns [6]. However, the limitations presented by this type of differential robot can be complemented by a different configuration, e.g., an Ackerman or an omnidirectional type. A robotic mechanism in the Ackerman configuration is the one found in conventional vehicles, where one motor drives the mobile, and another modifies its orientation. Although this configuration does not provide holonomicity to the mechanism, its application can solve tasks which require traction, since all the wheels have a fixed contact with the floor of the environment. On the other hand, the configuration, which allows moving the mobile in any of the directions, is known as omnidirectional. Depending on the type of wheels used, an omnidirectional robot can be placed at any point in the workspace while its orientation can be simultaneously modified [7,8]. In this way, the scalability of a set of cooperatively working robots can be maximized by using mobile robots of different characteristics, where the control methods can facilitate the inclusion of omnidirectional, differential, car-like, and so forth vehicles [9]. In the same context, heterogeneous robots can be used depending on the applications. Either they are working in structured or semi-structured environments, outdoor or indoor, and so on [10,11].

In related research, the concept of cooperation is generally interpreted in two different ways: Based on leader-follower and based on a virtual leader [12,13,14]. In the leader-follower consideration, one of the robotic mechanisms plays the role of leader complying with the tracking of the established path, while the other ones move relatively to the main robot. This configuration, although it is simple to implement, does not supply robustness when secondary objectives are required and depend directly on the execution of a single robot. In contrast, the virtual leader consideration generates a virtual formation that serves as a pattern for the rest of the vehicles to maintain a desired position and orientation. From a comparative perspective, the first consideration is led to applications where environmental disturbances are minimal, and computational processing is low [15]. On the other hand, the virtual leader approach allows executing secondary tasks such as obstacles avoidance, reshaping, minimizing of movements, and so on, since there is not a single robot in charge of achieving a control objective, but the formation follows a virtual leader, which can be modified due to disturbances in the environment. However, it requires a relatively high computational cost, as well as a resilient and robust communication structure. A cooperative robotics structure must additionally consider the distribution of control, which maintains the robotic set [16]. In the centralized control, one vehicle generates the calculation of all the velocities which the fellow robots must have as a reference to maintain the formation, where that robot has relatively high processing characteristics. On the contrary, the decentralized control considers that the formation characteristics are shared among the participants at a very high level, where the control actions to maintain the desired grouping are calculated for each vehicle individually. Although both configurations require some type of intercommunication to ensure satisfactory execution, the second case requires high reliability of data exchange to achieve the control objectives [17]. A considerable advantage of decentralized control is the integration of different control methods to the group of robots, taking into account that generally for cooperative robotics, the most important aspect is to maintain the position of the robotic set into a trajectory or path.

Depending on the hardware characteristics of the robotic systems, the controllers can be led to the optimization of processing or the fulfillment of the control objectives with the least possible error. In this way, the formation control can be implemented by various methods. For instance, [18] claims that some control methods are able to meet both the formation of a set of robots and to generate the reference velocities for each of the systems. Likewise, [19] proposes a control method for tracking trajectories based on numerical methods. The work presents a double manipulator embedded on a mobile platform, where the point of interest is in the middle of the two operational ends. The asymptotic stability of the control method is demonstrated analytically, where the experimental tests validate the execution of the formation. Similarly, a control method based on linear algebra is proposed by [20], where the kinematic system is approximated through linear algebra to find both the particular solution and the homogeneous solution, allowing to meet secondary objectives [21,22]. Experimental results are shown in order to demonstrate the stability of the proposed method. Another control method is mentioned in [23], where a cooperative control for non-holonomic robots is proposed through a finite-time controller. As the main contribution, the work is led to solve the finite-time consensus problem for multiple high-order nonholonomic mobile robots from a finite-time consensus control algorithm. Through simulation, it is shown that the controller can achieve the objectives in all the agents in the proposed finite time since the convergence of the method used is tested through a suitable Lyapunov function. In addition to the presented methods, [24] implements a pair of algorithms, the first based on a reinforcement learning algorithm and the second based on a particle filter. The main objective of the work is searching and tracking people with cooperative mobile robots, which locate goals, calculate their distance to them, and follow them, considering they work in highly unstructured spaces. The results of the proposed control algorithms are validated through an extensive set of simulations considering up to five agents, in addition to the hours of real-life experiments with two social robots.

Furthermore, the control methods for the formation of robots have a similar criterion as for a traditional robotic system, where the way in which the points of interest of all the participants are unified differs in many works. In this aspect, [25] can be considered, where the aim is controlling both the center and the angles of rotation and elevation of the triangle formed by a robotic set, a group that is greater than or equal to three members. The triangle formed supposes to be an irregular object held up by the operative end of all the mobile manipulators, where additionally, the redundancy of the systems is considered to carry out the evasion of obstacles or other secondary objectives. In contrast, the shape and orientation of the whole set can be configured through projections between each robot. Reference [26] takes into account this consideration, where the objective is to transport an object by two mobile anthropomorphic robots. The proposed controller to carry out the problem is based on the Lyapunov method, where it is analytically demonstrated that it is asymptotically stable. Additionally, experimental results are shown where three-dimensional platforms are used in order to evaluate the performance of the proposal.

In this work, the fulfillment of the primary and secondary objectives of a set of robots with heterogeneous characteristics and different modes of control and operation, working cooperatively, is analysed assuming that the position of each robot, the path to follow, and the shape of the formations are known as the initial condition, while taking into account that the robotic set knows the environment from external information or previously stored data. Through the proposal of a control algorithm based on linear algebra, which makes the computational cost less and becomes easily programmable, that are a great advantage in the implementation of the system, the set of robots can accomplish a task in a coordinated way by following a desired path as the primary task, while the formation of them is altered depending on the environment or the mission requirements (secondary tasks); this is the main novelty of the proposed system. Different to traditional ways of controlling a group of terrestrial robots, in this work the robotic set is moved along a path with a desired velocity not parameterized in time, which can depend on various purposes (evasion of obstacles encountered along the way, optimization of energy, and so on), while simultaneously it can alter the formation of the whole set through the modification of distances and orientations of the main and secondary projections. Contrary to works presented in [2,3,5,6], the formation scheme of heterogeneous robots is analyzed from projections between a pair of robots (being physical or virtual ones depending on the requirements), providing flexibility and scalability at the moment of adding up the robotic systems. Likewise, the controller based on linear algebra is designed, simulated, and implemented on the set of heterogeneous robots, further analyzing their stability. Another great advantage of the proposed system is that being a set of heterogeneous robots that operate, makes the kinematic and dynamic control of each residing in them, thus reducing the processing load of the system and giving the possibility of increasing robots of any operational feature, including their kinematic and dynamic control in it without the need to modify any other part of the system at all. It is not known of any work that has done something similar. Compared to references [8,9,14], the proposed controller uses a hierarchical control structure, in order to provide scalability to the system and at the same time, not saturate the processing unit, thus merging both the centralized and decentralized information processors. At a very high hierarchy level, a centralized computer is responsible for generating the control actions to achieve the primary and secondary objectives, while, at a local level, each member of the heterogeneous robotic set includes its own processing unity to achieve kinematic and dynamic control and also to provide feedback of odometric information, which is shared through wireless communications based on a data frame (assuming no constrains given by the environment). Additionally, another relevant advantage of the proposal is the low computational cost obtained through the control method based on linear algebra, avoiding saturating both the centralized computer and those ones located in each robot.

In [21] and many more, Gustavo Scaglia presents works based on Linear Algebra theories for collaborative control of mobile robots focused on the leading-follower technique; however, he does not consider the scalability of the robots nor the execution of the secondary control objectives, i.e., modification of robot formation, and obstacle avoidance, among other criteria. We, in our work, have done all of this, which makes it a substantial improvement.

This current work is presented in six sections, including the introduction and conclusions. Thus, Section 2 presents the multilayer control scheme, superficially describing the proposed control structure, while Section 3 presents the development of controllers based on numerical methods considering secondary tasks. Section 4 presents the controllers to execute the primary task, and the secondary task raised, analyzing the stability of both proposed methods. Section 5 shows the proposed kinematic control based on numerical methods, as well as a dynamic compensator for each robotic system. Finally, Section 6 shows three real experimental results to validate the proposed controllers, denoting the control responses of both the formation and each of the robots.

## 2. Multilayer Control Scheme

**Figure 1:**
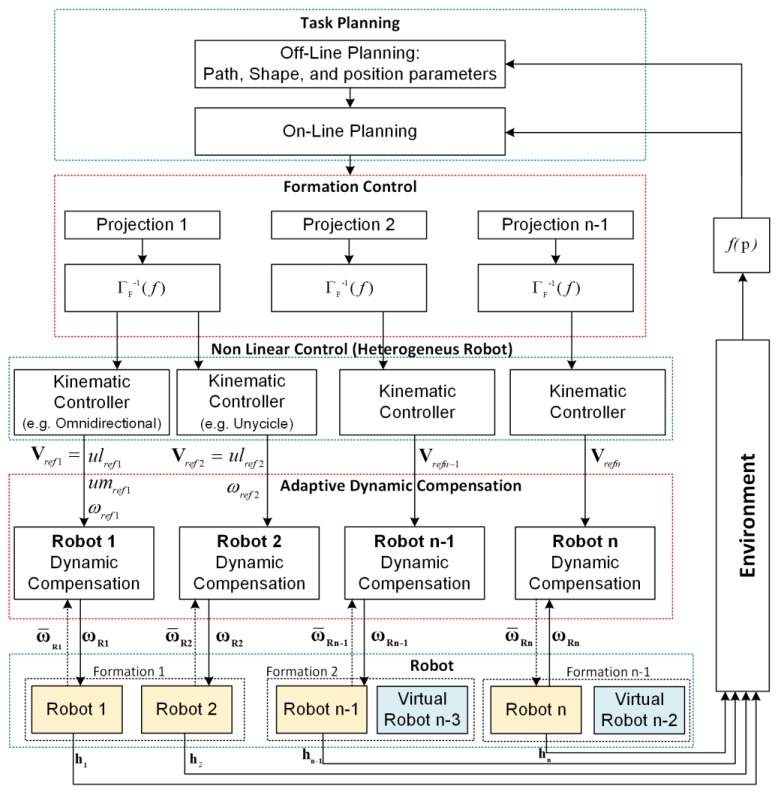
Multilayer control scheme.

The multilayer scheme defines how the appropriate variables that make up the cooperative control of heterogeneous mobile robots are distributed (Figure 1). Taking into account that each of the layers has different functionalities, the scheme is divided into six sections: (1) Acquisition of the characteristics of the environment allows planning the path to follow, maintaining the integrity of the robotic systems as well as fulfilling the pre-programmed task. This section has two layers, and in the off-line planning layer, the initial parameters are configured, i.e., the desired path to be followed, the starting locations of all the robotic systems, and the desired structure of formation. The on-line planning layer allows the execution of the secondary objectives, reacting to the unforeseen environmental changes in the off-line layer, produced by the lack of space to fulfill the desired parameters of formation, objects to avoid along the path, environmental conditions that require to change the formation performed, among other conditions, that can alter the fulfillment of the task. This is also very important because, through this scheme, you can navigate between the structured and unstructured environments. When the path is known, or the operating environment has already been obtained previously, this information is entered in this layer, if not, and as the robots are multi-sensors, you can easily install a radar and make the path planning and follow those trajectories found in real-time.

The objective of the (2) formation control layer is focused on calculating the control actions so that the vehicles maintain a positioning that satisfies the desired formation, generating the control signals for each of the robots. The (3) kinematic control layer takes as a reference the velocities generated by the formation control (upper level), i.e., the local controllers receive reference velocities to follow the profile guided by the formation, so these controllers are responsible for delivering maneuverability velocities that correspond of each heterogeneous robot involved (omnidirectional, car-like, unicycle). The (4) dynamics of each robot acting as an individual mechanism is solved by the adaptive dynamic compensation layer, within the control and operation scheme this layer is of paramount importance and provides, as mentioned above, the advantage that if robots of other characteristics are increased, each one has its own controller on top, which means that the only major modification that has to be made to the system is to enter the data from this robot into the communications frame, completely eliminating any change outside of this. The (5) Robots layer represents the set of heterogeneous robots considered in the formation. In addition, two virtual robots are used for the calculation of each of the projections. Finally, the (6) environment layer represents the structural space where the robot interacts, considering obstacles and other physical objects.

From what has been described, it can be noted that the work presented by us differs from the collaborative control literature in the following items: (1) Proposal of a modular multilayer control scheme, that is, it is possible to implement different control techniques without modifying the general structure of the collaborative control between n robots; (2) the control scheme considers both the centralized control technique, and the decentralized control technique, which allows generalization of the collaborative control to n heterogeneous robots; (3) the proposed control algorithms are based on the theory of linear algebra, which has as an advantage a low computational cost and, therefore, can be implemented in robots built with low-cost technology. In addition, when considering the linear algebra technique for the design of control algorithms, it is possible to incorporate redundancy in the transformation matrices of the formation, thus it is feasible to execute the main task (path follow-up of predefined tasks) and several secondary tasks, for example, modification of the formation, evasion of obstacles, fewer movements, among other control criteria; (4) the proposed algorithm considers path follow-up, that is, following the desired profile without being parameterized in time. Most of the works in the literature perform trajectory tracking, which is not feasible to implement in real applications since the trajectory tracking defines the desired position at a certain time. That is, it has a tracking profile parameterized in time; and finally (5) in the article presented there is a formal analysis of the stability of each one of the proposed control algorithms, in which mathematically asymptotic stability is demonstrated, that is, the control errors tend to zero.

## 3. Kinematic Transformation

Since the formations are composed of projections between a pair of robots, an additional formation is necessary to include a third robot, requiring the use of a virtual robot to generate the additional projection. It is assumed that the object to be transported or the shape that the robotic set should have does not have a defined geometry—it may have an unequal weight distribution, or it could be moving in a semi-structured space. In this aspect, the fulfillment of tasks with this type of objective can present better results if the robotic mechanisms have heterogeneous characteristics such as car-like, omnidirectional, or differential robots. The location of each of the robots depends totally on the requirements of the tasks, being able to alter the configurations at any moment of time if the workspace requires it.

**Figure 2:**
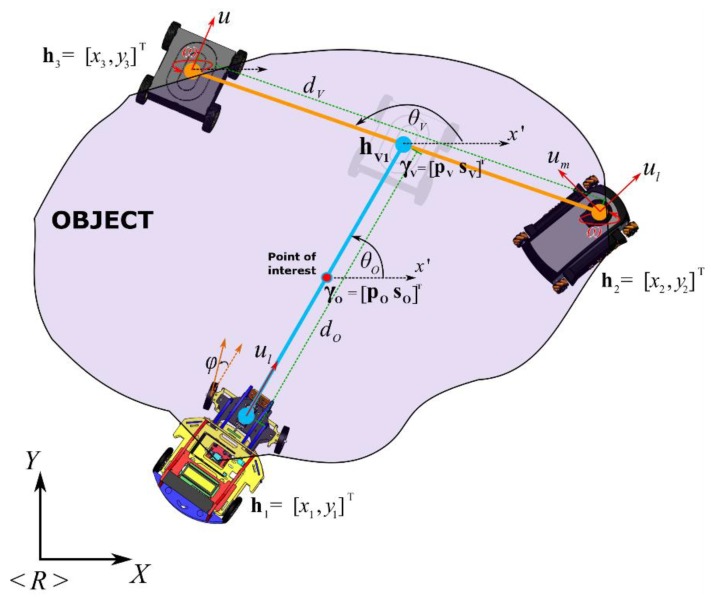
Formation analysis of heterogeneous robots.

Based on the formation presented for the analysis (Figure 2), the main projection to transport an object by three mobile vehicles is described as $\gamma_{O}={\left[\begin{array}{cc} p_{O} & s_{O} \end{array}\right]}^{T}\in {\mathbb{R}}^{4}$, where $p_{O}={\left[x_{O}y_{O}\right]}^{T}\in {\mathbb{R}}^{2}$ represents the position of the main intermediate point and $s_{O}={\left[\begin{array}{cc} \theta_{O} & d_{O} \end{array}\right]}^{T}\in {\mathbb{R}}^{2}$ indicates the shape of the projection, both with respect to the axis of reference 〈R〉. Thus, $p_{O}$ is located between the position of the first physical robot $h_{1}$ and the first virtual robot $h_{V1}$. On the other hand, for the second projection $\gamma_{V}={\left[\begin{array}{cc} p_{V} & s_{V} \end{array}\right]}^{T}\in {\mathbb{R}}^{4}$, the point $p_{V}={\left[x_{V}y_{V}\right]}^{T}\in {\mathbb{R}}^{2}$ is located between the position of the second $h_{2}$ and the third physical robot $h_{3}$ (the point which is also represented as the virtual robot $h_{V1}$), while $s_{V}={\left[\begin{array}{cc} d_{V} & \theta_{V} \end{array}\right]}^{T}$ describes the secondary shape. The characteristics of the transported object are defined by the distances and the desired angles formed between each of the robots, defined by $s_{O}={\left[d_{O}\theta_{O}\right]}^{T}$ and $s_{V}={\left[d_{V}\theta_{V}\right]}^{T}$, where $d_{O}$ represents the separation between the first robot and the virtual position, $d_{V}$ represents the separation between the other two physical robots $h_{2}$ and $h_{3}$, while $\theta_{O}$ and $\theta_{V}$ represent the orientation of both projections with respect to Y-axis and X-axis from the reference plane <R>.

The relationship between the formation pose-shape of each projection and the point-of-interest positions of each of the vehicles is given by the direct and inverse kinematic transformation, i.e., $\gamma_{k}=f\left(g_{k}\right)$ and $g_{k}=f^{-1}\left(\gamma_{k}\right)$, where $\gamma_{k}={\left[\begin{array}{cc} p_{k} & s_{k} \end{array}\right]}^{T}$ and $g_{k}={\left[\begin{array}{cc} h_{1st}^{T} & h_{2nd}^{T} \end{array}\right]}^{T}$, with k=O,V (main and secondary formations, respectively) for this case, while $h_{1st}^{T}$ and $h_{2nd}^{T}$ are the first and second robots of the projection, respectively. The direct kinematic transformations f(.) for both projections are given by:

(1) $$\gamma_{V}=\left[\begin{array}{c} p_{V}\\ s_{V} \end{array}\right];\;p_{V}=\left[\begin{array}{c} x_{V}\\ y_{V} \end{array}\right]=\left[\begin{array}{c} \frac{1}{2}\left(x_{3}+x_{2}\right)\\ \frac{1}{2}\left(y_{3}+y_{2}\right) \end{array}\right];\;s_{V}=\left[\begin{array}{c} d_{V}\\ \theta_{V} \end{array}\right]=\left[\begin{array}{c} \sqrt{{\left(x_{3}-x_{2}\right)}^{2}+{\left(y_{3}-y_{2}\right)}^{2}}\\ \tan^{-1}\left(\frac{y_{3}-y_{2}}{x_{3}-x_{2}}\right) \end{array}\right],$$

(2) $$\gamma_{O}=\left[\begin{array}{c} p_{O}\\ s_{O} \end{array}\right];\;p_{O}=\left[\begin{array}{c} x_{O}\\ y_{O} \end{array}\right]=\left[\begin{array}{c} \frac{1}{2}\left(x_{V}+x_{1}\right)\\ \frac{1}{2}\left(y_{V}+y_{1}\right) \end{array}\right];\;s_{O}=\left[\begin{array}{c} d_{O}\\ \theta_{O} \end{array}\right]=\left[\begin{array}{c} \sqrt{{\left(x_{V}-x_{1}\right)}^{2}+{\left(y_{V}-y_{1}\right)}^{2}}\\ \tan^{-1}\left(\frac{y_{V}-y_{1}}{x_{V}-x_{1}}\right) \end{array}\right].$$

In turn, the inverse kinematic transformation f(.) is denoted by

(3) $$g_{V}=\left[\begin{array}{c} h_{3}\\ h_{2} \end{array}\right]=\left[\begin{array}{c} x_{V}+\frac{1}{2}d_{V}\cos\theta_{V}\\ y_{V}+\frac{1}{2}d_{V}\sin\theta_{V}\\ x_{V}-\frac{1}{2}d_{V}\cos\theta_{V}\\ y_{V}-\frac{1}{2}d_{V}\sin\theta_{V} \end{array}\right]\;\mathrm{and}$$

(4) $$g_{O}=\left[\begin{array}{c} p_{V}\\ h_{1} \end{array}\right]=\left[\begin{array}{c} x_{O}+\frac{1}{2}d_{O}\cos\theta_{O}\\ y_{O}+\frac{1}{2}d_{O}\sin\theta_{O}\\ x_{O}-\frac{1}{2}d_{O}\cos\theta_{O}\\ y_{O}-\frac{1}{2}d_{O}\sin\theta_{O} \end{array}\right]$$

**Remark** **1:** $x_{O},y_{O},x_{V},y_{V}$ *are located on the frame of the inertial reference.*

The relationship between the variation of g(t) and γ(t) can be obtained through the time derivative of the forward and the inverse kinematics transformations, represented by the Jacobian matrix $\Gamma_{F}\left(g\right)$ defined as:

(5) $$\dot{\gamma }\left(t\right)=\Gamma_{F}\left(g\right)\dot{g}\left(t\right)$$

and in the inverse way is given by:

(6) $$\dot{g}\left(t\right)=\Gamma_{F}^{-1}\left(\gamma \right)\dot{\gamma }\left(t\right),$$

where

$$\Gamma_{F}\left(g\right)=\frac{\partial \gamma_{fx1}}{\partial g_{\mathrm{ex}1}}\;\mathrm{and}\;\Gamma_{F}^{-1}\left(\gamma \right)=\frac{\partial g_{\mathrm{ex}1}}{\partial \gamma_{fx1}}\;\mathrm{with}\;e,f=4$$.

## 4. Controllers Design

### 4.1. Controller Analysis Considering Secondary Objectives

The design of the kinematic controllers proposed in this paper was based on numerical methods tools. To facilitate the search for the solution of a set of equations, a system can be represented in a matrix structure, where theorems and axioms of linear algebra are applied. In this way, it is considered the first-order differential equation:

(7) $$\dot{\eta }\left(t\right)=f\left(\eta \left(t\right),\dot{\zeta }\left(t\right)\right),$$

where η(t) represents the output of the system to be the controller with initial conditions $\eta \left(0\right)=\eta_{0}$, $\dot{\eta }\left(t\right)$ is the first derivative with respect to time, and $\dot{\zeta }\left(t\right)$ is the control action. Furthermore, η(t) becomes η(k) in the discrete time with $t=kT_{0}$, where $T_{0}$ represents the proposed sampling time respecting the Nyquist theorem, and k are the samples of the continuous response.

Given that the state and the control action on the time instant t(k−1) are known, the system’s state at instant t(k) can be approximated by Euler’s method [27] as

(8) $$\frac{\eta \left(k\right)-\eta \left(k-1\right)}{T_{0}}=f\left(\eta \left(t\right),\dot{\zeta }\left(t\right)\right).$$

The design of the kinematic controller was based on the kinematic transformation of the mobile vehicles cooperation. In order to design a formation controller or a path following, the kinematic transformation can be approximated as:

(9) $$\frac{\eta \left(k\right)-\eta \left(k-1\right)}{T_{0}}=\Gamma \left(\zeta \left(k\right)\right)\dot{\zeta }\left(k\right)$$

where Γ(ζ(k)) contains the characteristics of both the positioning and the formation of all the robotic systems in the case of the formation controller or the positioning information of the object in the case of the path following.

**Figure 3:**
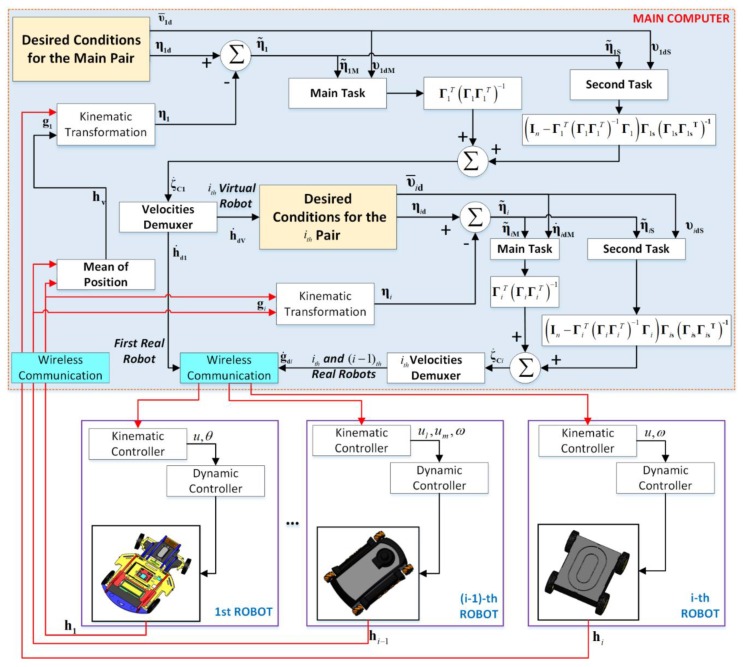
Implemented control structure.

It is important to mention that following a path consists of maintaining the position and orientation of the object to be transported within a predefined route without parameterization in time. In this way, the control objective is to position the object at the closest point of the marked path P at the desired velocity $\upsilon_{d}$. To reach this goal, the following expression is considered,

(10) $$\frac{\eta \left(k\right)-\eta \left(k-1\right)}{T_{0}}=\upsilon_{d}+\frac{W\left(\eta_{d}\left(k-1\right)-\eta \left(k-1\right)\right)}{T_{0}}$$

where $\eta_{d}\left(k-1\right)$ is a point of the desired path with the desired shape at the previous instant of k and $W\left(\tilde{\eta }\left(k-1\right)\right)$ is a diagonal matrix that weights control errors $\tilde{\eta }\left(k-1\right)=\eta_{d}\left(k-1\right)-\eta \left(k-1\right)$, defined as:

(11) $$W\left({\tilde{\eta }}_{m}\left(k-1\right)\right)=\frac{w_{m}}{1+\left|{\tilde{\eta }}_{m}\left(k-1\right)\right|},$$

with $\tilde{\eta }$ represents the error vector at the outputs of the controller (see Figure 3), and m that represents each of the states of formation of the cooperative control. Now, Equations (9) and (10) can be considered to generate the system of equations

(12) $$\Gamma \left(\zeta \left(k\right)\right)\dot{\zeta }\left(k\right)=\upsilon_{d}+\frac{W\left(\eta_{d}\left(k-1\right)-\eta \left(k-1\right)\right)}{T_{0}},$$

which let us rewrite the system as Au=b, where $A=\Gamma \left(\zeta \left(k\right)\right)\in {\mathbb{R}}^{m\times n}$, $u=\dot{\zeta }\left(k\right)\in {\mathbb{R}}^{m}$, and $b=\upsilon_{d}+\frac{W\left(\eta_{d}\left(k-1\right)-\eta \left(k-1\right)\right)}{T_{0}}$, with $b\in {\mathbb{R}}^{m}$. In this way, the control actions are defined as $u=A^{-1}b$. In case A is quadratic (with det(A)≠0), A has a direct inverse solution, otherwise, it must be necessary to use a method to solve the pseudoinverse problem, given by:

(13) $$\underset{u}{\underbrace{\dot{\zeta }\left(k\right)}}=\underset{A^{-1}}{\underbrace{\Gamma {\left(\zeta \left(k\right)\right)}^{T}{\left(\Gamma \left(\zeta \left(k\right)\right)\Gamma {\left(\zeta \left(k\right)\right)}^{T}\right)}^{-1}}}\underset{b}{\underbrace{\left({\dot{\eta }}_{d}\left(k\right)+\frac{W\left(\eta_{d}\left(k-1\right)-\eta \left(k-1\right)\right)}{T_{0}}\right)}}.$$

**Remark** **2:** *The pseudo-inverse here applied was that introduced by Moore and Penrose. It has the property that it thus distributes the coefficients of the redundant columns in the solution that the sum of squares of these coefficients was minimized [28]. Unfortunately, in cases where loses rank, it is known that the pseudo-inverse approach will not always avoid singular configurations. In singularities, the original task is replaced by a solution of which does not precisely correspond to the original one. By means of the use of the Gram-Schmidt algorithm or the Singular Value Decomposition, pseudoinverses can be constructed in which the singularities can be avoided, but that is beyond the scope of this work.*

Furthermore, it is said that a system of linear equations is homogeneous if it can be written in the form Au=0. Now, assuming that the configuration of the robotic system (13) is redundant, the Jacobian matrix $\Gamma \left(\zeta \left(k\right)\right)\in {\mathbb{R}}^{m\times n}$ has more unknowns than equations (m<n) with range r=n for each b, and taking into account that the homogenous equation has a not trivial solution, the system could have infinite solutions. In this case, suppose the equation Au=b is consistent for a b given and letting $v_{p}$ be a particular solution, the solution is the set of all the vectors of the form

(14) $$v=v_{p}+v_{h},$$

with $v_{h}$ as any solution of the homogeneous system $\Gamma \left(\zeta \left(k\right)\right)v_{h}=0$.

A viable solution method is to formulate the problem as a constrained linear optimization problem

(15) $$\frac{1}{2}{\left\|v\right\|}_{2}{}^{2}=min,$$

yielding the particular solution

(16) $$v_{p}=\Gamma {\left(\zeta \left(k\right)\right)}^{T}{\left(\Gamma \left(\zeta \left(k\right)\right)\Gamma {\left(\zeta \left(k\right)\right)}^{T}\right)}^{-1}b.$$

On the other hand, the null space of Γ(ζ(k)) in ${\mathbb{R}}^{n}$ is the set of velocities that do not produce any effect over the actions of the heterogeneous robotic set. In that case, it is necessary to rethink the cost function, expressing it as

(17) $$\frac{1}{2}\|v-v_{0}\|{{}_{2}}^{2}=min.$$

that yields the homogeneous solution

(18) $$v_{h}=\left(I_{n}-\Gamma {\left(\zeta \left(k\right)\right)}^{T}{\left(\Gamma \left(\zeta \left(k\right)\right)\Gamma {\left(\zeta \left(k\right)\right)}^{T}\right)}^{-1}\Gamma \left(\zeta \left(k\right)\right)\right)v_{0}.$$

Thereby, inserting Equations (16) and (18) into (14), it yields the proposed law of control

(19) $$v_{c}=\underset{v_{p}}{\underbrace{\Gamma {\left(\zeta \left(k\right)\right)}^{T}{\left(\Gamma \left(\zeta \left(k\right)\right)\Gamma {\left(\zeta \left(k\right)\right)}^{T}\right)}^{-1}b}}+\underset{v_{h}}{\underbrace{\left(I_{n}-\Gamma {\left(\zeta \left(k\right)\right)}^{T}{\left(\Gamma \left(\zeta \left(k\right)\right)\Gamma {\left(\zeta \left(k\right)\right)}^{T}\right)}^{-1}\Gamma \left(\zeta \left(k\right)\right)\right)v_{0}}},$$

where the first term on the left-hand side is the particular solution $v_{p}$ and second term $v_{h}$ of this equation belong to the null space of Γ(ζ(k)).

For the purposes of this work, the second term in Equation (19) represents the projection on the null space of the robotic systems, where $v_{0}$ is an arbitrary vector that contains the velocities associated with the shape and orientations of the robotic set. Therefore, any value given to $v_{0}$ will affect the internal structure of the formation only, but not the final control of the first objective at all. The null space created by the high-level task matrix allows each velocity to be projected onto that space, where the sub-tasks compete to solve the problem in different ways. However, the velocities of the second task need to be calculated and included in $v_{0}$, being

(20) $$v_{0}=\Gamma_{s}\left(\zeta \left(k\right)\right){\left(\Gamma_{s}\left(\zeta \left(k\right)\right)\Gamma_{s}{\left(\zeta \left(k\right)\right)}^{T}\right)}^{-1}\left({\dot{\eta }}_{d}\left(k\right)+\frac{W_{S}\left(\eta_{dS}\left(k-1\right)-\eta_{S}\left(k-1\right)\right)}{T_{0}}\right),$$

where $\Gamma_{s}\left(\zeta \left(k\right)\right)$ is the Jacobian matrix, which contains the secondary objectives.

**Remark** **3:** *The consideration of having more than one robot working on the same purpose allows achieving secondary tasks at the same time collectively. To achieve these objectives, a behavior-based controller can be implemented to split tasks into sub-objectives, solving problems separately, and finally combining them to obtain the final solution.*

### 4.2. Formation and Following Control

The cooperative control of heterogeneous mobiles considers that the type of robots incorporated into the formation system includes its own kinematic controller and a dynamic compensator, therefore, for the formation control, the type of system included is transparent. This section briefly describes the basic control scheme to maintain the formation of many robotic systems, a basis to integrate more robots in a scalable way. Figure 3 shows the control structure implemented for this work, denoting both the controllers which maintain the formation, orientation, and following of the desired path and the controllers that correct the final actions of each robot.

The adoption of the null-space approach was due to the possibility of treating tasks and subtasks separately, which were unified at the end to get the control actions. Since the main task does not conflict with the secondary task, the designer of the controllers selected the task hierarchy. By means of this, different control objectives can be achieved, e.g., maximum manipulability, energy saving, obstacle avoidance, and so on [29]. In this case, the main task was to reach that the set of robots followed a path, while the secondary task was to maintain the geometric shape and orientation of every projection formed by the vehicles.

The unified Jacobian matrix in Equation (9) contains both the first derivatives corresponding to the positions of the center point of a formation, as well as the distance and the orientation between the two robots forming the projection. For the calculation of different control actions, it is taken into account that the Jacobian matrix in Equation (9) is divided into two parts:

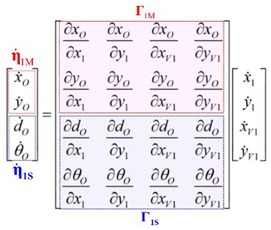
(21)
where $\Gamma_{1M}\left(\zeta \left(k\right)\right)\in R^{2\times 4}$ are the first derivatives of the midpoint positions of the projection, $\Gamma_{1S}\left(\zeta \left(k\right)\right)\in R^{2\times 4}$ are the first derivatives of the distance and orientation angle, ${\dot{\eta }}_{1M}\in R^{2\times 1}$ corresponds to the first variable to be calculated and ${\dot{\eta }}_{1S}\in R^{2\times 1}$ the second variable to calculate.

**Figure 4:**
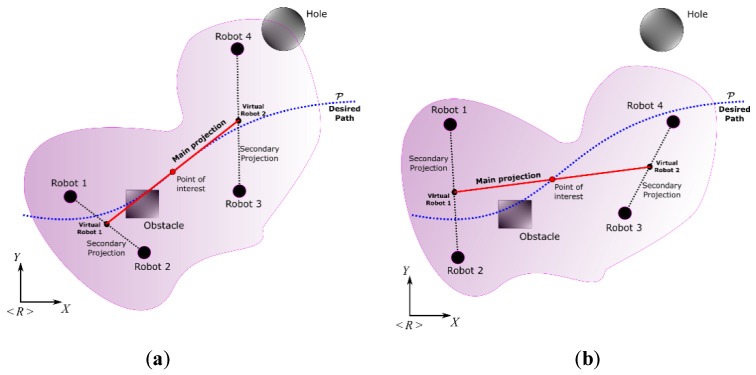
Control possibilities considering the path following and formation of the robotic set.

Considering the proposed splitting in Equation (21), differentiated controls can be proposed to achieve both the primary and secondary objectives. The work proposes as the main objective of the point of interest $p_{O}$ (redefined as $\eta_{1M}$ for the application of the proposed controller) follows a profile not parameterized in time, with a velocity $\upsilon_{d}$ imposed by the designer. On the other hand, the secondary objective was to modify the formation of robotic systems, as long as they did not conflict with satisfying the first objective. Having a formation based on projections, the main projection $\gamma_{O}$ plays an important role since it is the basis for calculating the velocities that the rest of the projections will adopt to fulfill the tasks. Additionally, having a main straight line that can be projected and oriented according to the requirements of the controller designer allows locating the robotic systems to be agreed upon. Contrast the possibilities (a) and (b) of Figure 4; the first projection has a different length and orientation to the second projection. However, the main objective was achieved in both, where the variation in distances and secondary orientations did not conflict with the main task. The following paragraphs describe the characteristics mentioned in more detail.

(a) Path following controller

To achieve that the center of the projection $\eta_{iM}$ follows the desired path, the proposed controller is defined by

(22) $${\dot{\zeta }}_{iM}\left(k\right)=\Gamma_{iM}{\left(\zeta \left(k\right)\right)}^{T}{\left(\Gamma_{iM}\left(\zeta \left(k\right)\right)\Gamma_{iM}{\left(\zeta \left(k\right)\right)}^{T}\right)}^{-1}\left({\overset{-}{\upsilon }}_{idM}+W_{iM}\left({\tilde{\eta }}_{iM}\left(k-1\right)\right)\right),$$

where $\Gamma_{iM}(\zeta \left(k\right))$ is the first part of the general Jacobian that determines the characteristics of the robotic set to follow the desired path, with ${\tilde{\eta }}_{iM}\left(k-1\right)=\eta_{diM}\left(k-1\right)-\eta_{iM}\left(k-1\right)$. The obtained response in ${\dot{\zeta }}_{iM}\left(k\right)$ are two pairs of velocities that will be applied to the robots located at the ends of the projection, whether physical or virtual. These velocities are due to maintain the point of interest in the desired path but leaving aside the errors of the distance between the robots and the angle formed with respect to 〈R〉. It is important to note that the main projection (where the point of interest of the transported object is located) can have the desired velocity, in this case, ${\overset{-}{\upsilon }}_{idM}=\upsilon_{dM}$, while the rest of projections will have velocities generated by the main controller, i.e., ${\overset{-}{\upsilon }}_{idM}={\dot{\eta }}_{idM}$.

(b) Formation and Guidance Control

This part of the control allows the establishment of the desired formation $\eta_{iS}$ as a secondary objective, providing flexibility to projection modifications between the robots in applications where the tasks of the tracking targets were required, e.g., given the presence of the obstacles on the road. The second part of the Jacobian matrix is considered in this controller, which provides the characteristics of formation and orientation $\Gamma_{iS}\left(\zeta \left(k\right)\right)$ of both robotic systems. In this way, the controller is defined by

(23) $${\dot{\zeta }}_{2}\left(k\right)=\Gamma_{iS}{\left(\zeta \left(k\right)\right)}^{T}{\left(\Gamma_{iS}\left(\zeta \left(k\right)\right)\Gamma_{iS}{\left(\zeta \left(k\right)\right)}^{T}\right)}^{-1}\left(\upsilon_{dS}+W_{2}\left({\tilde{\eta }}_{iS}\left(k-1\right)\right)\right).$$

Contrary to a), this controller aims to correct the shape of the $i_{th}$ projection, zeroing distance errors between the robots, and general orientation of the robotic pair.

(c) Unified Controller

This section defines the priority in the control tasks that the group of robots will have. The work proposes the positioning control of the point of interest (in this case, the midpoint of the main projection). Therefore, the task that does not conflict with the main control objective is the form and orientation of the projection. Based on (19), the proposed final controller is presented in the form

(24) $$\begin{array}{l} {\dot{\zeta }}_{C}\left(k\right)=\Gamma_{iM}{\left(\zeta \right)}^{T}{\left(\Gamma_{iM}\left(\zeta \right)\Gamma_{iM}{\left(\zeta \right)}^{T}\right)}^{-1}\left({\overset{-}{\upsilon }}_{idM}+\frac{W\left({\tilde{\eta }}_{iM}\left(k-1\right)\right)}{T_{0}}\right)+\\ \left(I_{n}-\Gamma_{iM}{\left(\zeta \right)}^{T}{\left(\Gamma_{iM}\left(\zeta \right)\Gamma_{iM}{\left(\zeta \right)}^{T}\right)}^{-1}\Gamma_{iM}\left(\zeta \right)\right)\Gamma_{iS}\left(\zeta \right){\left(\Gamma_{iS}\left(\zeta \right)\Gamma_{iS}{\left(\zeta \right)}^{T}\right)}^{-1}\left(\upsilon_{idS}+\frac{W_{S}\left({\tilde{\eta }}_{iS}\left(k-1\right)\right)}{T_{0}}\right), \end{array}$$

where the second part of this controller shows the attempt to correct the errors so long as it does not interfere with the main task, which is carried out by the first part of the controller. This fusion allows having the desired velocities for the pair of robots that form the $i_{th}$ projection.

**Remark** **4:** *Cases (a), (b), and (c) are applicable to one of the* i *possible projections, requiring* i *controllers to be programmed as projections exist. This facilitates the scalability of the system, while at the same time making the robotics set more flexible by giving desired parameters to each of the projections at any time.*

**Remark** **5:** *In the case study,*$\upsilon_{idS}$*can be imposed by the controller designer, but it must be directly related to the error reduction, i.e., a velocity can be configured with which the shape is corrected to desired parameters, but it must have a null value in the event the objectives are achieved, otherwise, errors caused by the same correction action can appear. One way to solve this is to force it to be immersed within the weight given by*$W_{S}$.

(d) Stability Analysis

Rewriting the proposed control law (24) in a way

(25) $${\dot{\zeta }}_{C}\left(k\right)=\Gamma_{M}^{\#}\left({\overset{-}{\upsilon }}_{dM}+\frac{W_{M}\left({\tilde{\eta }}_{M}\left(k-1\right)\right)}{T_{0}}\right)+\left(I-\Gamma_{M}^{\#}\Gamma_{M}\right)\Gamma_{S}^{\#}\left(\upsilon_{dS}+\frac{W_{S}\left({\tilde{\eta }}_{S}\left(k-1\right)\right)}{T_{0}}\right),$$

where $\Gamma_{M}^{\#}=\Gamma_{M}{\left(\zeta \right)}^{T}{\left(\Gamma_{M}\left(\zeta \right)\Gamma_{M}{\left(\zeta \right)}^{T}\right)}^{-1}$ and $\Gamma_{S}^{\#}=\Gamma_{S}{\left(\zeta \right)}^{T}{\left(\Gamma_{S}\left(\zeta \right)\Gamma_{S}{\left(\zeta \right)}^{T}\right)}^{-1}$ represent a pseudo-inverse matrix notation, the analysis can be developed in a simpler manner.

Now, the analysis is done for the control of the main objective, in addition, it is considered that the secondary objective of the formation is not affected when Equation (25) is multiplied by $\Gamma_{M}$.

(26) $$\begin{array}{cc} \Gamma_{M}{\dot{\zeta }}_{C}\left(k\right)=\Gamma_{M} & \Gamma_{M}^{\#}\left({\overset{-}{\upsilon }}_{dM}+\frac{W_{M}\left({\tilde{\eta }}_{M}\left(k-1\right)\right)}{T_{0}}\right)\\ & +\Gamma_{M}\left(I-\Gamma_{M}^{\#}\Gamma_{M}\right)\Gamma_{S}^{\#}\left(\upsilon_{dS}+\frac{W_{S}\left({\tilde{\eta }}_{S}\left(k-1\right)\right)}{T_{0}}\right) \end{array}$$

then, as $\Gamma_{M}\Gamma_{M}^{\#}=I$ and $\Gamma_{M}\left(I-\Gamma_{M}^{\#}\Gamma_{M}\right)=0$, Equation (26) is reduced to

(27) $$\Gamma_{M}{\dot{\zeta }}_{C}\left(k\right)=\left({\overset{-}{\upsilon }}_{dM}+\frac{W_{M}\left({\tilde{\eta }}_{M}\left(k-1\right)\right)}{T_{0}}\right)$$

The stability analysis for Equation (27) is done by means of the feedback, i.e., if it is considered that Equation (21) be perfect tracking then, ${\dot{\eta }}_{M}\left(k\right)≅\Gamma_{M}{\dot{\zeta }}_{C}\left(k\right)$, and taking into account that the error for the primary objective is defined as $\eta_{dM}\left(k-1\right)-\eta_{M}\left(k-1\right)$ such that

(28) $${\dot{\eta }}_{M}\left(k\right)={\overset{-}{\upsilon }}_{dM}+W_{M}\left(\frac{\eta_{dM}\left(k-1\right)-\eta_{M}\left(k-1\right)}{T_{0}}\right);$$

therefore, replacing the velocity for the main objective ${\dot{\eta }}_{M}\left(k\right)=\frac{\Delta {\dot{\eta }}_{M}\left(k\right)}{T_{0}}=\frac{\eta_{M}\left(k\right)-\eta_{M}\left(k-1\right)}{T_{0}}$ in Equation (28) and also considering that the desired velocity for the path is ${\overset{-}{\upsilon }}_{dM}={\dot{\eta }}_{dM}-{\dot{\gamma }}_{M}$:

(29) $$\frac{\eta_{M}\left(k\right)-\eta_{M}\left(k-1\right)}{T_{0}}={\dot{\eta }}_{dM}-{\dot{\gamma }}_{M}+W_{M}\left(\frac{\eta_{dM}\left(k-1\right)-\eta_{M}\left(k-1\right)}{T_{0}}\right).$$

Next, the differential derivative is applied to obtain ${\dot{\eta }}_{dM}\left(k\right)=\frac{\eta_{dM}\left(k\right)-\eta_{dM}\left(k-1\right)}{T_{0}}$ and the same way to ${\dot{\gamma }}_{M}\left(k\right)=\frac{\Delta \gamma_{M}}{T_{0}}$, then the expression

(30) $$\frac{\eta_{M}\left(k\right)-\eta_{M}\left(k-1\right)}{T_{0}}=\frac{\eta_{dM}\left(k\right)-\eta_{dM}\left(k-1\right)}{T_{0}}-\frac{\Delta \gamma_{M}}{T_{0}}+W_{M}\left(\frac{\eta_{dM}\left(k-1\right)-\eta_{M}\left(k-1\right)}{T_{0}}\right)$$

Simplifying $T_{0}$ and grouping Equation (30) with the current states (k) and the previous state (k−1) there is

(31) $$\begin{array}{ll} (\eta_{dM}\left(k-1\right)- & \eta_{M}\left(k-1\right))\\ & =\left(\eta_{dM}\left(k\right)-\eta_{M}\left(k\right)\right)-\Delta \gamma_{M}+W_{M}\left(\eta_{dM}\left(k-1\right)-\eta_{M}\left(k-1\right)\right) \end{array}$$

Now, taking account that $\eta_{d_{M}}k-\eta_{M}k={\tilde{\eta }}_{M}k$ and $\eta_{d_{M}}\left(k-1\right)-\eta_{M}\left(k-1\right)={\tilde{\eta }}_{M}\left(k-1\right)$ represent the current error and the previous error, respectively, Equation (31) is reduced to

(32) $${\tilde{\eta }}_{M}\left(k-1\right)={\tilde{\eta }}_{M}\left(k\right)-\Delta \gamma_{M}+W_{M}\left({\tilde{\eta }}_{M}\left(k-1\right)\right),$$

and grouping similar terms, it results that

(33) $$\Delta \gamma_{M}={\tilde{\eta }}_{M}\left(k\right)+W_{M}\left({\tilde{\eta }}_{M}\left(k-1\right)\right)-{\tilde{\eta }}_{M}\left(k-1\right).$$

The primary objective error ${\tilde{\eta }}_{M}\left(k\right)$ is separated to have the following expression

(34) $$\Delta \gamma_{M}={\tilde{\eta }}_{M}\left(k\right)+{\tilde{\eta }}_{M}\left(k-1\right)\left(W_{M}-I\right),$$

therefore, stability in discrete time is realized by the *Z* transform, and taking account the analysis for each diagonal matrix value $W_{M}$ so

(35) $$\left(1-z^{-1}\right)\gamma_{M}\left(z\right)={\tilde{\eta }}_{M}\left(z\right)+{\tilde{\eta }}_{M}\left(z\right)z^{-1}\left(W_{M}-I\right)\;\tilde{\eta }\left(z\right)=\frac{1-z^{-1}}{1+z^{-1}\left(W_{M}-I\right)}\gamma \left(z\right).$$

Finally, the poles of the function obtained in the following way are analyzed:

$$1+z^{-1}\left(W_{M}-1\right)=0$$

(36) $$z=1-W_{M}$$

Thus, for the control to be stable, the *Z* plane pole must be inside the unit circle. Therefore, the values of the diagonal matrix must be considered as $0<diag\left(W_{M}\right)<1$, hence the control error ${\tilde{\eta }}_{M}\left(k\right)$ come close to being zero when k→∞, then the system is asymptotically stable.

Now, as a second step, we proceed to effectuate the analysis for the secondary objective of formation. In a similar way to the primary objective, multiply both members of the control law by $\Gamma_{S}$, then Equation (25) it is written as:

(37) $$\Gamma_{S}{\dot{\zeta }}_{C}\left(k\right)=\Gamma_{S}\Gamma_{M}^{\#}\left({\overset{-}{\upsilon }}_{dM}+\frac{W_{M}\left({\tilde{\eta }}_{M}\left(k-1\right)\right)}{T_{0}}\right)+\Gamma_{S}\left(I-\Gamma_{M}^{\#}\Gamma_{M}\right)\Gamma_{S}^{\#}\left(\upsilon_{dS}+\frac{W_{S}\left({\tilde{\eta }}_{S}\left(k-1\right)\right)}{T_{0}}\right).$$

According to [28], where an important property of Jacobian matrices is analyzed when one does not have a conflict between them, i.e., that it is feasible to fulfill the two tasks simultaneously and completely, it results that

(38) $$\Gamma_{S}\Gamma_{M}^{\#}=0.$$

Therefore, if Equations (36) in (37) are replaced, the below expression is obtained:

(39) $$\Gamma_{S}{\dot{\zeta }}_{C}\left(k\right)=\Gamma_{S}\Gamma_{S}^{\#}\left(\upsilon_{dS}+\frac{W_{S}\left({\tilde{\eta }}_{S}\left(k-1\right)\right)}{T_{0}}\right).$$

Additionally, considering Equation (21) to be perfect tracking, there is ${\dot{\eta }}_{S}\left(k\right)≅\Gamma_{S}{\dot{\zeta }}_{C}\left(k\right)$. Performing a similar procedure of analysis that Equations (27) in (39), it is concluded in the same way that $z=I-W_{S}$, therefore the values of the diagonal matrix must be considered as $0<diag\left(W_{S}\right)<1$, hence the control error ${\tilde{\eta }}_{S}\left(k\right)$ come close to being zero when k→∞, then the system is asymptotically stable.

## 5. Kinematics Control and Dynamic Compensation of Each Robot

The velocities delivered by the position and formation controller are inputs for each of the robots, where the local controllers interpret independently the velocities as the profile ${\dot{h}}_{i}$ to follow. This section describes the control of each of the heterogeneous robots that could be considered in the set, both for the kinematic control and for the correction of errors given by the dynamics of each system.

(a) Kinematics control

The kinematic model of a mobile robot gives the location of a point of interest to any part of the robotic system. The instantaneous kinematic model of a mobile robot gives the derivative of its point of interest as a function of the derivatives of the whole system itself

(40) $${\dot{h}}_{i}\left(t\right)=J_{i}\left(q\right)v_{i}\left(t\right),$$

where ${\dot{h}}_{i}={\left[\begin{array}{cc} {\dot{h}}_{xi} & {\dot{h}}_{yi} \end{array}\right]}^{T}$ is the vector of the point of interest velocities, $v_{i}={\left[\begin{array}{ccc} u_{li} & u_{mi} & \omega_{i} \end{array}\ldots \right]}^{T}$ is the vector of the mobile robot velocities, which contains the linear and angular velocities of the mobile platform, $J_{i}\left(q\right)$ is the Jacobian matrix, which defines a linear mapping between the vector of the mobile robot velocities $v_{i}\left(t\right)$ and the vector of the point of interest velocities, and i is the $i_{th}$ robot of the set.

Through the Euler’s approximation of the kinematic model of whatever mobile robot, the following kinematic model discrete is obtained by:

(41) $$h\left(k+1\right)=h\left(k\right)+T_{0}J\left(q\left(k\right)\right)v\left(k\right),$$

where, values of h at the discrete time $t=kT_{0}$ will be denoted as h(k), with $T_{0}$ as the sample time, and k∈{0,1,2,3,…}. Next, by the Markov property and to adjusting the performance of the proposed control law, the states vector h(k+1) are replaced by:

(42) $$h\left(k+1\right)=h_{d}\left(k+1\right)-W\left(h_{d}\left(k\right)-h\left(k\right)\right),$$

where W is a diagonal matrix, which must satisfy $0<diag\left(W_{hx},W_{hy}\right)<1$, allowing to reduce the variations in state variables, and $h_{d}$ is the desired trajectory.

Then, from Equations (41) and (42), the following system of linear equations is obtained, which allows at each sampling instant to calculate the control actions

(43) J(q)v=b,

where $v={\left[\begin{array}{ccc} u_{l}\left(k\right) & u_{m}\left(k\right) & \omega \left(k\right) \end{array}\right]}^{T}$ and:

(44) $$b=\frac{1}{T_{0}}\left[\begin{array}{c} h_{xd}\left(k+1\right)-w_{hx}\left(e_{hx}\left(k\right)\right)-h_{x}\left(k\right)\\ h_{yd}\left(k+1\right)-w_{hy}\left(e_{hy}\left(k\right)\right)-h_{y}\left(k\right) \end{array}\right]$$

From Equation (43), its solution by least squares is obtained by solving the normal equations

(45) $$v_{ref}=J{\left(q\right)}^{T}{\left(J\left(q\right)J{\left(q\right)}^{T}\right)}^{-1}\frac{1}{T_{0}}\left[\begin{array}{c} h_{xd}\left(k+1\right)-w_{hx}\left(e_{hx}\left(k\right)\right)-h_{x}\left(k\right)\\ h_{yd}\left(k+1\right)-w_{hy}\left(e_{hy}\left(k\right)\right)-h_{y}\left(k\right) \end{array}\right]$$

(b) Dynamics compensation

This section details how the controller was designed to compensate the robot’s dynamics in the workspace, achieving greater robustness for each robot. A dynamics controller was programed within each mobile platform, i.e., robotic platforms have an internal control that corrects linear and angular velocity errors.

**Figure 5:**
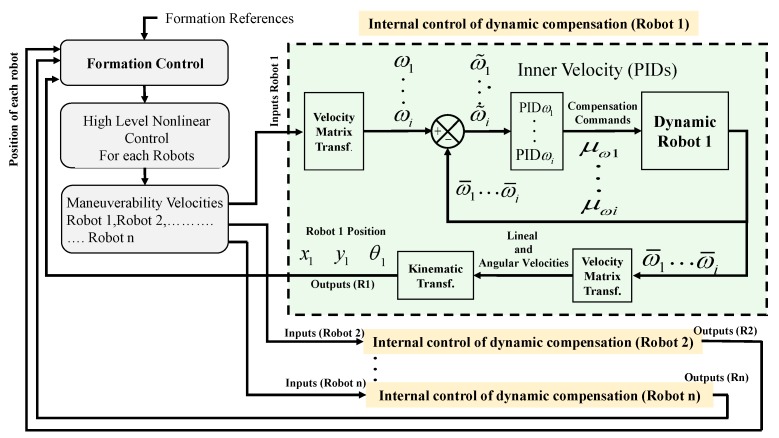
Control operation scheme for dynamic compensation.

Linear or angular errors can be caused by external forces or torques that affect the dynamics of the platforms. Given that all the robots received the maneuverability commands as input (linear and angular ones), the number of velocities depended on each mobile robot to meet the formation requirements. Figure 5 describes how the internal controller is structured to compensate for the dynamics of robots. In this case, the controller generates a control output defined as:

(46) $$\mu_{\omega i}\left(k\right)=Kp_{i}\left\{{\tilde{\omega }}_{i}\left(k\right)+Ki_{i}T_{o}\sum_{j=1}^{k}{\tilde{\omega }}_{i}\left(j-1\right)+K_{D}{}_{i}\frac{{\tilde{\omega }}_{i}\left(k\right)-{\tilde{\omega }}_{i}\left(k-1\right)}{T_{o}}\right\},$$

where, ${\tilde{\omega }}_{i}\left(k\right)=\omega_{i}\left(k\right)-{\overset{-}{\omega }}_{i}\left(k\right)$ represents the angular velocity error between the desired angular velocity $\omega_{i}\left(k\right)$ and the actual angular velocity ${\overset{-}{\omega }}_{i}\left(k\right)$ with i=1, 2, 3,… (i depends on the number of controlled traction wheels of each robot), the variables $Kp_{i},Ki_{i},K_{D}{}_{i}$ are the gains that weigh the control error, and $T_{o}$ is the sampling time considered for the discrete PID (Proportional, Integral and Derivative control). Direct and reverse transformation matrices depend on the configuration of each mobile robot to achieve linear velocities, angular, and, eventually, the position and orientation of the mobile robot to feedback the control loop of the proposed cooperative scheme.

## 6. Robots Instrumentation

### 6.1. Sensors for Relative Position

Within the multiple robotic systems, electronic components were used to power the embedded processing units, know the state of the charge of the batteries, share information with remote receivers wirelessly, know the odometry of the system, and so on. Some of these sensors provided information on the state of each robotic system, allowing the robot to locate itself within their workspace in order to avoid collisions. The robots used for the experiments of this work have built-in sensors to estimate the position given a relative location, i.e., the wheels of each of the robots contain encoders that measure the velocity and the direction of rotation. These sensors determined the angular displacement, and thus the linear displacement of the robots, showing limitations when there were landslides or the resolution of the encoders was not adequate. The proposed control algorithm considers the knowledge of the current position of each one of the mobile robots at all times of the task; therefore, when starting the desired task, the initial condition was the position and orientation of each robot that was part of the collaborative task.

### 6.2. Communication System

**Figure 6:**
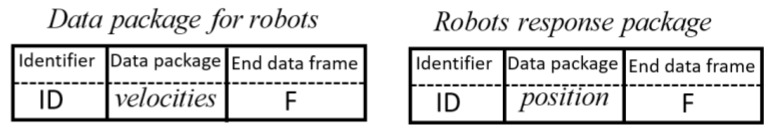
Master-slave data package frame.

The communication structure proposed for the set of robots was based on the IEEE 802.15.4 protocol. This protocol allows the creation of multipoint networks, and it is also capable of transmitting a large amount of data (bytes) with very low latency and achieving a predictable communication synchronization. The XBEE S2 modules allow the implementation of the established protocol, configured in such a way that they are all within the same wireless network. The topology used in this work was the mesh configuration, i.e., all devices can communicate with each other in such a way that each robot has an identifier to know which data package corresponds to it. This provides the advantage if, at some instant of time the communication fails or is lost, communication can continue between all the other nodes (mobile robots) because the connectivity is forced to be restored. The coordinator of the network sends the maneuverability commands to each robot (linear velocities and angular velocities), where the velocities are packaged within a data frame. Figure 6 shows how the data packet sent by the master-robot and the package that responds to the slave-robots.

**Figure 7:**
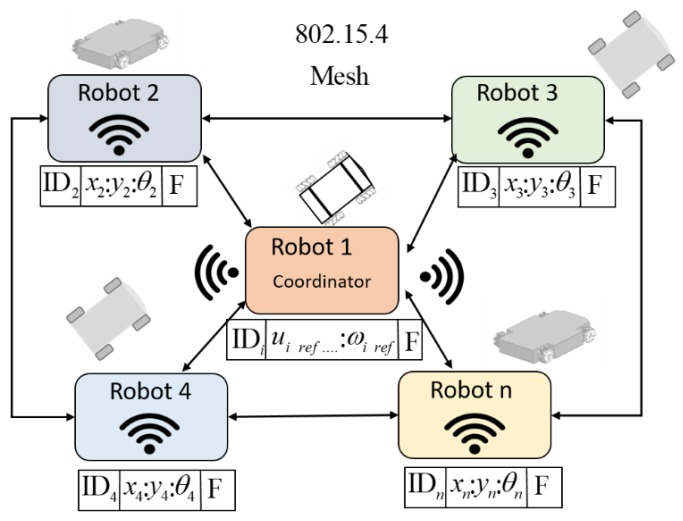
The mesh structure of communication used.

The proposed communication scheme complies with a sampling rate of 175 ms, i.e., send-receive and process data from robots within the proposed network. Figure 7 shows how the communication among the robots is structured into the cooperative system. Communications in this type of MESH configuration can eventually be made between 65,000 devices without losing their characteristics, according to the manufacturers data. In this work, the cost of increasing robots makes the frames larger, and that implies a reduction in the velocity of communications, which is established can not be less than 100 ms. Increasing the number of robots that exceed this margin would imply an application of a massive use of robots, but if this were the case, the restriction of communications in the network would have to be increased, reducing the velocity of the system itself: e.g., in an application of object transport, the whole would move at lower velocities.

**Remark** **6:** *The encoding and size of packets sent to each robot depend on the type of mobile platform, while the feedback information has similar sizes. As a MESH configuration was used in this work, the robots were able to exchange all the information between them, whether they were the destination or not; they received it to process it or only as a rebroadcast node. This means that the robots exchange all the information of the system as transport nodes but only process the information of which they are the addressees.*

## 7. Experimental Results

**Figure 8:**
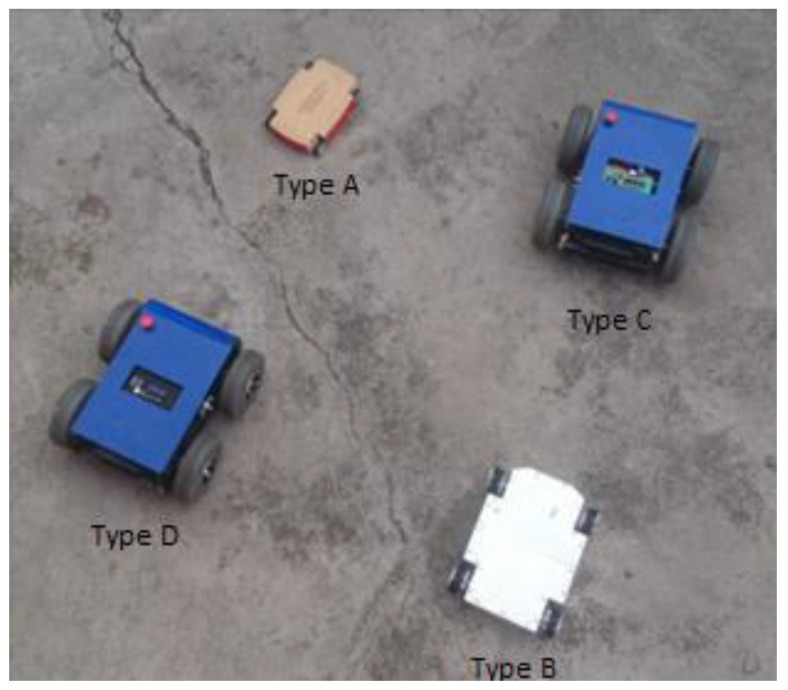
Robots considered for experimental results.

Experimental results are shown in this Section. To demonstrate the scalability of the proposed system, the fulfillment of tasks with two, three, and four robots were proposed. In order to include more robots, it was necessary to add the recognition of communication frames and a control segment for the new projection. At the computational level, this did not significantly increase the processing time since dynamic and kinematic controllers were carried out in each robot. An additional simulated result was included at the end, in order to validate the scalability of the control system. The control scheme presented in Figure 3 allows meeting the objectives of shape and position for a system of multiple heterogeneous robots. For the validation of the proposed controller, robots of different dynamic configurations were used in order to experience the scalability of the cooperative control. Figure 8 shows the robots used for the experiments performed. It can be seen that the set of robots was heterogeneous, with two holonomic omnidirectional robots of different characteristics: Type A was an omnidirectional robot, while the Type B robot was very similar but contained components of greater performance that made it able to carry heavy weights; the Type C robot was a normal unicycle robot, with two rear-wheel drive, and finally the Type D robot had a four-wheel drive.

### 7.1. First Experiment

**Figure 9:**
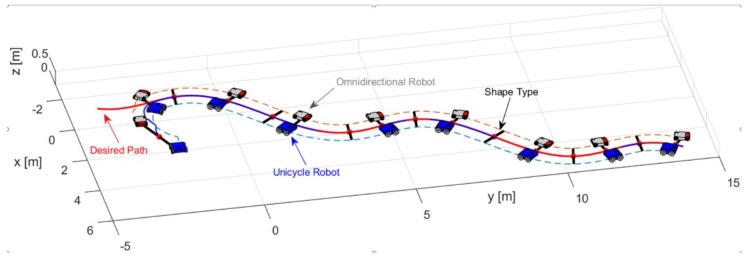
Movement executed by the robots.

The first experiment consisted of executing a formation between a unicycle robot (Type C robot) and an omnidirectional robot (Type A robot). The desired path ρ(s) is described by $x_{dO}=\frac{4}{5}\cos\left(\frac{27}{100}t\right)+\frac{13}{100}t-2\;\left[m\right]$, $y_{dO}=\frac{3}{10}t-4\;\left[m\right]$, during 40 [sec] with $\upsilon_{d}=0.2\;\left[\frac{m}{s}\right]$; take into account that the desired path may be the result of an offline path planning or planned online. The formation parameters defined for the experiment are: $d_{O}=1\;\left[m\right]$ and $\theta_{O}=\frac{\pi }{2}+\tan^{-1}\left(\frac{{\dot{y}}_{dO}}{{\dot{x}}_{dO}}\right)\;\left[rad\right]$. Figure 9 illustrates the path executed by the robots, where the primary objective $\left(x_{O},y_{O}\right)$ stays on the desired path. On the other hand, the second objective during the experiment meets the shape defined, i.e., orientation and distance.

**Figure 10:**
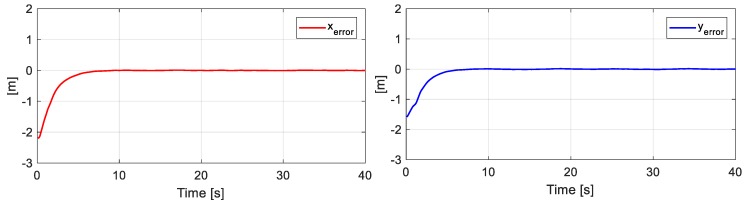
Errors of the main objective.

Figure 10 presents the desired position errors $\tilde{\eta }\left(k\right)$ of the object. It is noted that at the beginning of the experiment, there was a major error that was caused by the initial positions of each robot. Once the point closest to the path was found, the mobile platforms were positioned in such a way that the position of the main objective was on the desired path.

**Figure 11:**
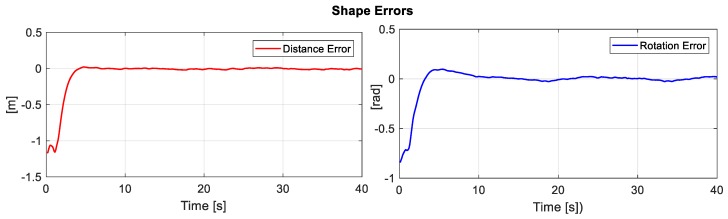
Errors of the secondary objective (distance and orientation).

Figure 11 displays the error of the secondary objective, i.e., the distance error and angle error of formation between both the robots.

**Figure 12:**
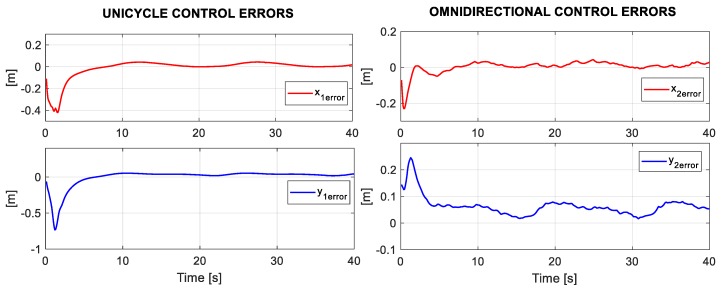
Position errors of the Unicycle and Omnidirectional robots during the experiment.

For the planned experiment, the formation controls generated velocities references $\left[\begin{array}{cc} {\dot{h}}_{1} & {\dot{h}}_{2} \end{array}\right]$, which by means of the kinematic control, each robot led to follow a trajectory to fulfill the formation expected. Therefore, in Figure 12, the position errors of each robot during the experiment are presented.

### 7.2. Second Experiment

**Figure 13:**
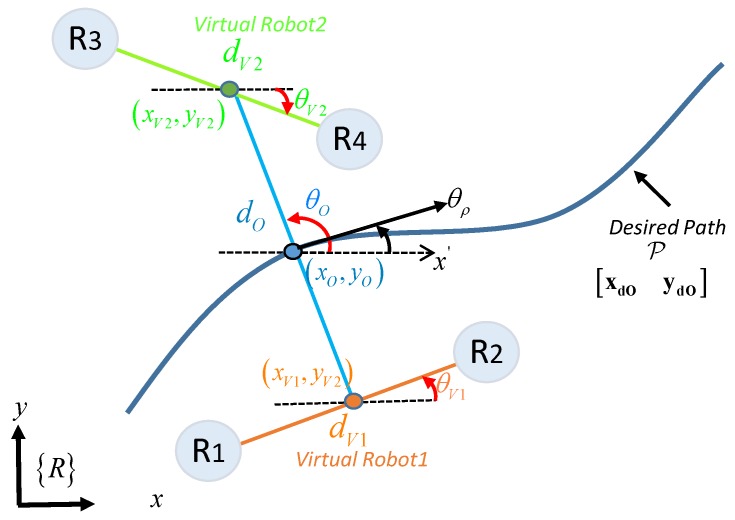
Formation parameters scheme for the second experiment.

The second experiment propounds to incorporate four robots for the formation, two unicycle-type mobile platforms (Type C and D robot), and two omnidirectional-type mobile platforms (Type A and B robot). In contrast, with the previous experiment, the desired formation for the four robots varied the shape parameters during the experimentation, the change in shape consisted of varying the angle and the defined virtual distances. Figure 13 indicates the shape parameters for the proposed cooperative control experiment, the number of parameters depends on the type of shape and the number of robots incorporated. Table 1 indicates the parameters desired for this experiment.

**Figure 14:**
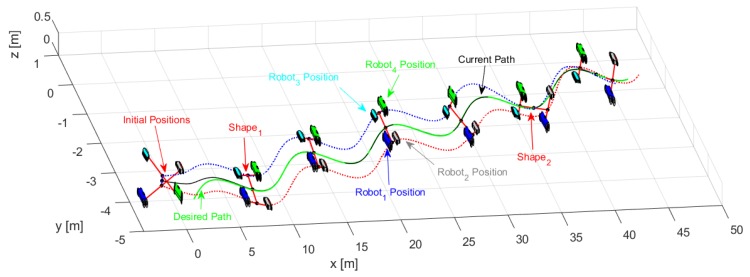
Robots movement to complete the task.

**Figure 15:**
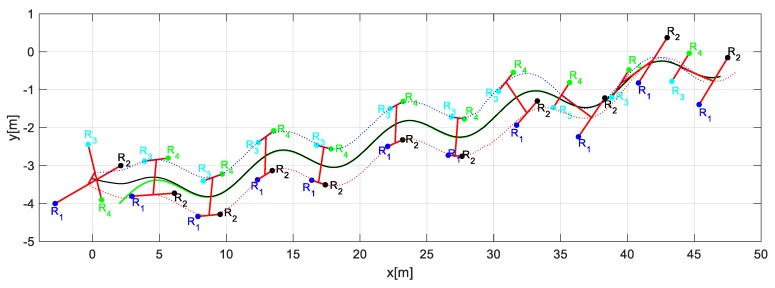
Stroboscopic movement of the second experiment.

Through the changing of shape, it was verified that during the execution of the task, the robots could autonomously modify the positions to move the object from one place to another following a defined path. Figure 14 shows the stroboscopic movement of the executed task, while in Figure 15, it is appreciated how the shape of the robotic set changes gradually.

**Figure 16:**
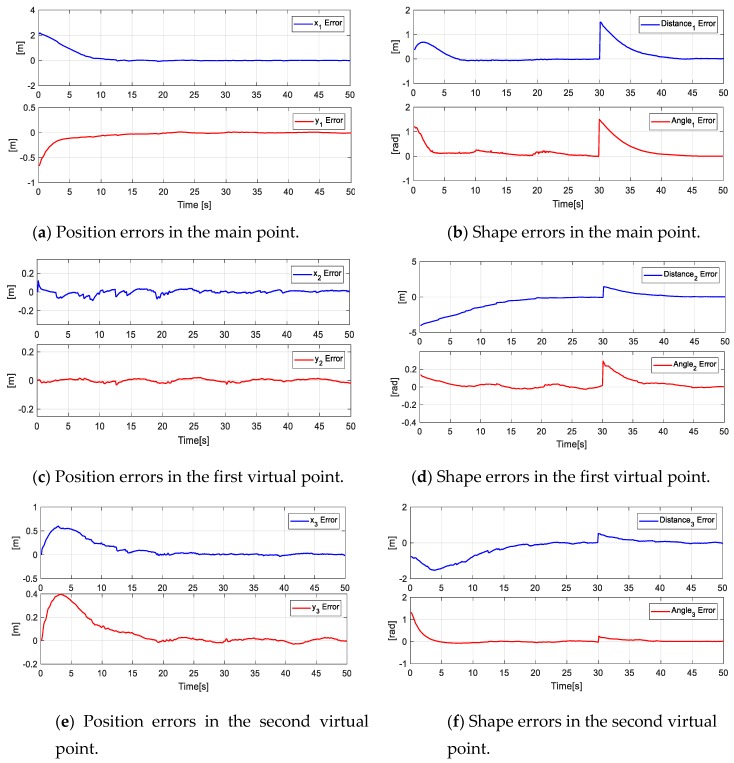
Main Objective errors and secondary objective errors at the primary point and virtual points of the formation.

Figure 16 shows the errors of the formation (main objective - secondary objectives), i.e., the errors of the main point $\left[{\tilde{x}}_{O},{\tilde{y}}_{O},{\tilde{d}}_{O},{\tilde{\theta }}_{O}\right]$. In the same way, the errors of virtual parameters are denoted as $\left[{\tilde{x}}_{V1},{\tilde{y}}_{V1},{\tilde{d}}_{V1},{\tilde{\theta }}_{V1},{\tilde{x}}_{V2},{\tilde{y}}_{V2},{\tilde{d}}_{V2},{\tilde{\theta }}_{V2}\right]$.

**Figure 17:**
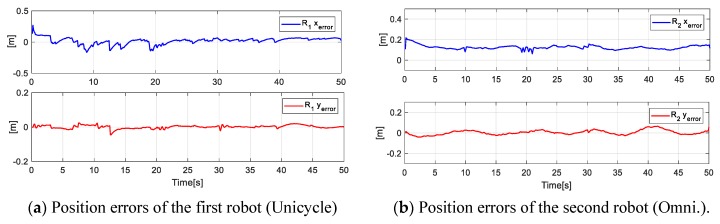

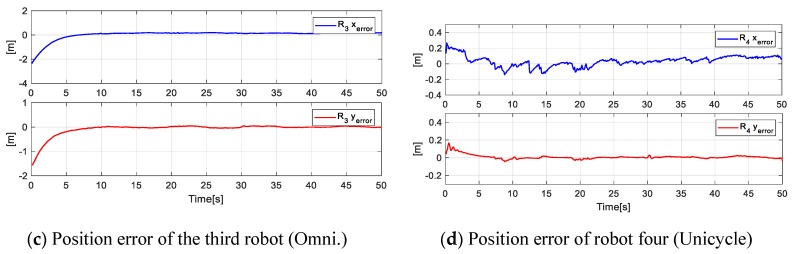
Position errors of each of the four robots for the desired shape.

Finally, Figure 17 indicates the position errors of each robot during the experimentation, where it is shown that the error is approaching zero of each robot in the executed experimentation.

### 7.3. Third Experiment

**Figure 18:**
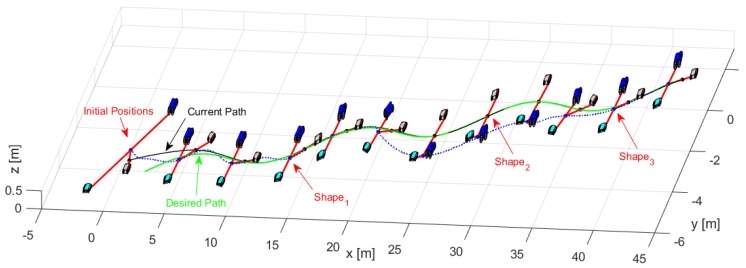
Evolution movement by the robots for the third experiment.

The third experiment was performed with three robots (Type C-D-A robots) changing the formation conditions of distances and orientations at an instant of time t. Table 2 indicates the desired parameters for the formation. Figure 18 shows how the robots comply with the requirements of position and shape.

**Figure 19:**
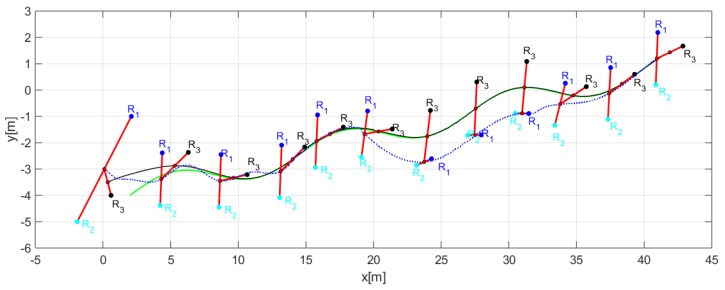
Position of each robot to complete the desired shapes.

Figure 19, shows in detail the formation of robots as they follow the desired path, demonstrating that each robot changed the position according to the required shape.

**Figure 20:**
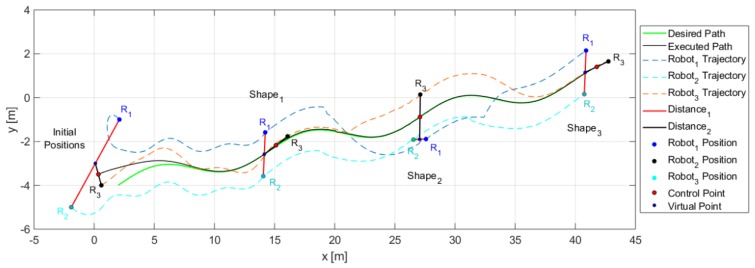
Robot positions and trajectories executed to stay on track with the desired shapes for the third experiment.

Figure 20 shows how the movement of each robot evolves to comply with the desired shapes.

**Figure 21:**
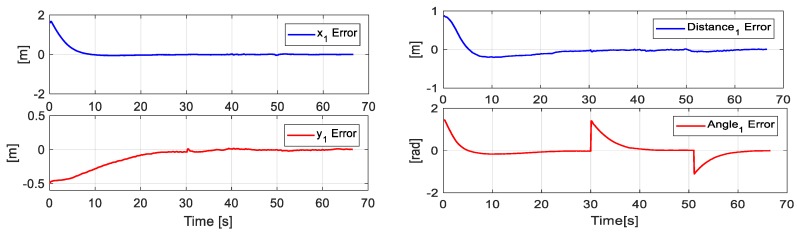

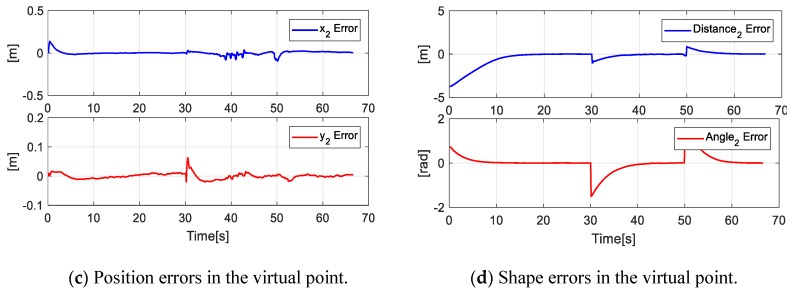
Errors of the main and second objectives in the third experiment.

To validate the correct operation of the controller and verify that the robots complied with the task designed in shape and position, the errors are shown in Figure 21.

### 7.4. Fourth Experiment

**Figure 22:**
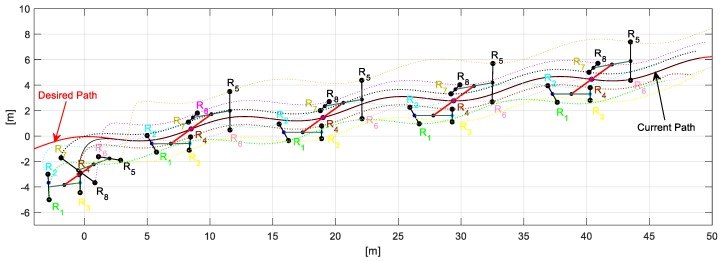
Robot positions and trajectories executed to stay on track with the desired shapes for the fourth experiment.

**Figure 23:**
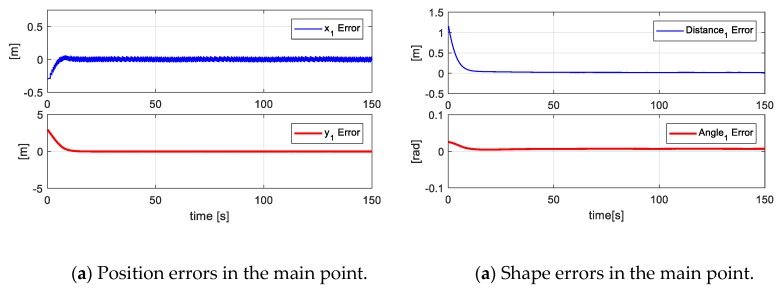

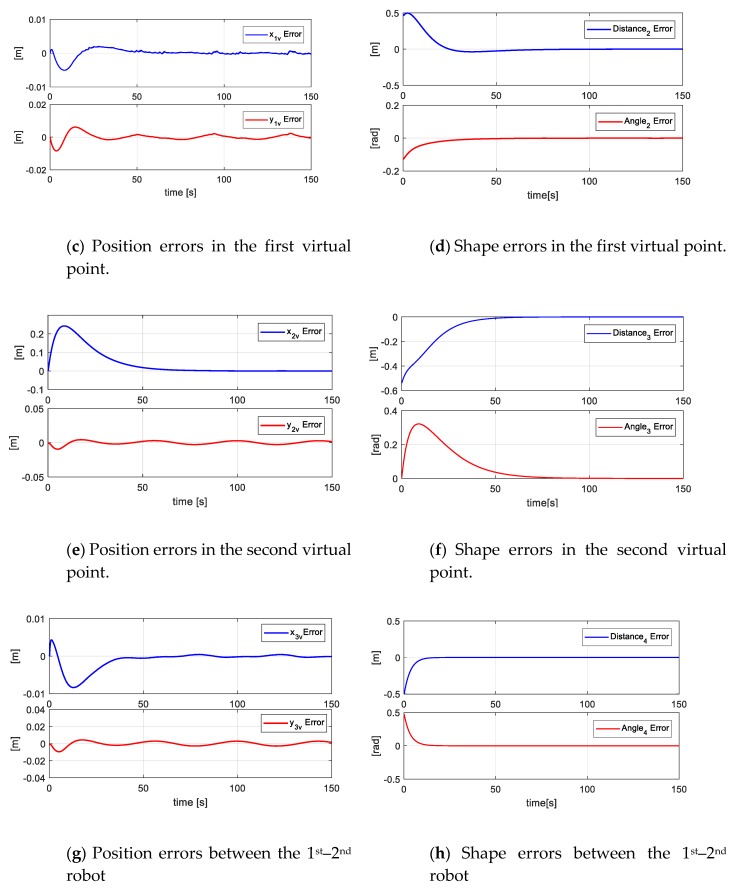
Errors of objectives in the fourth experiment.

In order to demonstrate the system scalability, this additional experiment was taken into account. For this, a task with eight robots is carried out in a simulated environment, given the hardware limitations. Figure 22 shows the stroboscopic movements of the robotic set, while Figure 23 presents the control errors of the first, second, third, and fourth projections. The rest of the projections are supposed to tend to zero, given the stroboscopic results.

## 8. Conclusions

Cooperative control considering primary and secondary objectives is achieved through linear algebra methods in this work, where diverse heterogeneous robots were used to determine the performance of the developed controllers. Compared with other formation control techniques, this proposal proposes to create projections for each robot that is included, providing the system with scalability in an easier way than other ones found in related works. Moreover, this work mathematically analyses the needed controllers to fulfill a primary task, in this case, the path followed by the robotic set, while the shape and orientation of all the robotic systems can be modified depending on the application without altering the main task given the redundancy of the system. Additionally, the stability analysis of the control law is presented to know the maximum values that the calibration parameters must have, as well as to demonstrate that the system is asymptotically stable. Furthermore, this work fuses centralized and decentralized processing of tasks in order to have a computer calculating the primary and secondary objectives, while processing units assure the kinematic and dynamic control of each robot. Finally, the set of robots used to obtain the experimental results is shown, results that demonstrate the proper functioning of the proposed controller through different real experiments where up to four different mechanisms are used. Everything described can be applied in civil construction environments, for transport and placement of materials in strategic sites; in warehouse management for transport of heavy objects of various characteristics and dimensions, and finally also in military applications for placement of dissuasive objectives in training camps.

## Figures and Tables

**Table 1:** Parameters formation for the experiment.

Formation Parameter	0>t<30 [s]	30≥t≤50 [s]
$\upsilon_{d}$	0.15 [m/s]	
$x_{dO}$	$\frac{3}{10}t+2\;\left[m\right]$	
$y_{dO}$	$\frac{2}{5}\sin\left(\frac{1}{5}t\right)+\frac{1}{40}t-4\;\left[m\right]$	
$\theta_{\rho }$	$\tan^{-1}\left(\frac{{\dot{y}}_{dO}}{{\dot{x}}_{dO}}\right)\;\left[rad\right]$	
$d_{O}$	1 [m]	2.5 [m]
$\theta_{O}$	$\theta_{\rho }+\frac{\pi }{2}\;\left[rad\right]$	$\tan^{-1}\left(\frac{{\dot{y}}_{dO}}{{\dot{x}}_{dO}}\right)\;\left[rad\right]$
$d_{V1}$	1 [m]	2.5 [m]
$\theta_{V1}$	$\tan^{-1}\left(\frac{{\dot{y}}_{dO}}{{\dot{x}}_{dO}}\right)\;\left[rad\right]$	$\frac{\pi }{6}\;\left[rad\right]$
$d_{V2}$	1 [m]	2 [m]
$\theta_{V2}$	$\tan^{-1}\left(\frac{{\dot{y}}_{dO}}{{\dot{x}}_{dO}}\right)\;\left[rad\right]$	$\frac{\pi }{6}\;\left[rad\right]$

**Table 2:** Formation parameters for the third experiment.

Formation Parameters	0>t<30 [s]	30≥t<50 [s]	50≥t≤70 [s]
$\upsilon_{d}$	0.17 [m/s]		
$x_{dO}$	$\frac{2}{5}t+2\;\left[m\right]$		
$y_{dO}$	$\frac{1}{2}\sin\left(\frac{1}{5}t\right)+\frac{1}{20}t-4\;\left[m\right]$		
$\theta_{\rho }$	$\tan^{-1}\left(\frac{{\dot{y}}_{dO}}{{\dot{x}}_{dO}}\right)\;\left[rad\right]$		
$d_{O}$	2 [m]	2 [m]	2 [m]
$\theta_{O}$	$\theta_{\rho }\;\left[rad\right]$	$\theta_{\rho }+\frac{\pi }{2}\;\left[rad\right]$	$\theta_{\rho }\;\left[rad\right]$
$d_{V1}$	2 [m]	1 [m]	2 [m]
$\theta_{V1}$	$\frac{\pi }{2}\;\left[rad\right]$	0 [rad]	$\frac{\pi }{2}\;\left[rad\right]$
